# Effect of topical *Areca palm* L. hydroalcoholic extract on burn wound healing in rats

**DOI:** 10.5455/javar.2021.h553

**Published:** 2021-11-02

**Authors:** Zahra Abbasy, Abolfazl Azami Tameh, Reza Mozaffari-Kermani, Hamid Zaferani Arani, Sayyed Alireza Talaei

**Affiliations:** 1 Faculty of Medicine, Kashan University of Medical Sciences, Kashan, Iran; 2 Anatomical Sciences Research Center, Institute for Basic Sciences, Kashan University of Medical Sciences, Kashan, Iran; 3 Department of Pathology, Tehran Medical Sciences, Islamic Azad University, Tehran, Iran; 4 Young Researchers and Elite Club, Tehran Medical Sciences, Islamic Azad University, Tehran, Iran; 5 Physiology Research Center, Institute for Basic Sciences, Kashan University of Medical Sciences, Kashan, Iran

**Keywords:** *Areca catechu*, hydroalcoholic extract, burn wound, wound healing, rats

## Abstract

**Objectives:**

Wound healing is a complex and dynamic process that begins immediately following tissue injury and continues until the wound is completely healed and remodeled. Applying the most effective burn repair techniques is a constant challenge in medicine. Antiulcerogenic and wound healing properties of *Areca palm* leaves have been validated through various investigations and animal studies. This study aimed to determine the potential for *A. palm* hydroalcoholic extract to heal burn wounds in rats.

**Materials and Methods:**

For 14 days, we examined 40 male Wistar albino rats in 5 groups: those receiving 1% silver sulfadiazine cream (reference standard), those receiving eucerin (positive control), and those receiving 5% and 10% ointments of *Areca catechu* hydroalcoholic extract (treatment groups). No treatment was given to the negative control group. On the dorsal part of the animals’ necks, burn wounds were made. After the rats were sacrificed, the wound contraction rate (WCR) was determined, and the wound sites were histopathologically examined.

**Results:**

On the 14th day, the WCR was significantly higher in rats treated with *A. palm* 10% extract ointment than in rats treated with 5% extract, positive or negative control groups (*p *< 0.001), or rats treated with silver sulphfadiazine (*p *= 0.01). After applying a 10% extract ointment to burn wound sites, complete healing occurred with only mild tissue inflammation and edema.

**Conclusion:**

The study’s findings indicate that the hydroalcoholic extract of *A. palm* L. has the ability to expedite the wound healing process. Additional research is necessary to identify the compounds responsible for their wound healing properties and comprehend their action mechanism.

## Introduction

Burn is one of the most common types of injuries worldwide, affecting approximately 1 million Americans each year, resulting in 40,000 hospitalizations and 3,400 deaths [1,2]. It could be caused by a variety of factors, including heat, electricity, chemicals, or radiation [3]. Burn wound severity is classified into first, second, and third degrees based on the depth and degree of skin damage [4]. On the other hand, Rehman et al. [5] reported that prolonged dehydration caused by a burn wound might result in a variety of morbidities such as acute kidney injury, septic shock, decreased liver function, or amputation.

Wound healing is a complicated and dynamic process that begins immediately following tissue injury and continues until the wound heals and remodels [6,7]. Applying the most effective burn repair techniques is a continuous challenge in medicine [3]. Despite the numerous chemical products used to treat burn wounds, worldwide interest in natural products and traditional medicine is growing [8]. Today, people believe that using herbal remedies with potent antioxidant and anti-inflammatory properties will accelerate wound healing [9].

*Areca catechu* L. is a slender palm in the Arecaceae family that grows abundantly throughout Asia, East Africa, and the Pacific. It is commonly referred to as betel nut [10,11]. The Areca tree’s leaves are green and contain catechin, tannins, gallic acid, fat, gum, and alkaloids, such as arecoline and arecaidine [11,12].

Recent research and animal studies demonstrate that *Areca palm* possesses a variety of therapeutic properties, including antibacterial [13,14], antifungal [14,15], antiparasitic [14,15], antioxidant [15,16], anti-inflammatory [15], analgesic [15], hepatoprotective [17], hypoglycemic [18], antiulcerogenic [19], wound healing [16], antidepressant [20], antifertility [21], abortifacient, and anti-implantation [22] effects. 

A study found that betel leaf contains an active mixture of essential oils (hydroxyl chavicol, eugenol, chavicol, chavibethol, estragol, terpene, sequiterpene, triterpenoid, and -cytosterol) and tannin [23]. According to Thi et al. [24], phytochemical compounds in betel leaf can induce an inflammatory response and stimulate the re-epithelialization process. However, to our knowledge, no such literature exists for the wound healing activity of the title plant’s leaves extract in the form of an ointment. Additionally, traditional medicines such as Ayurveda and Siddha use these leaves as a component of medicinal oils to treat burn wounds [25]. Vonna et al. [26] and Verma et al. [27] discovered that areca nut ethanol extract has wound healing properties in mice. According to our findings, the existing literature on the effect of topical *A. Palm* L. hydroalcoholic extract on burn wound healing is insufficient; thus, this study was conducted.

## Materials and Methods

### Ethical approval

The study protocol was approved by the Kashan University of Medical Sciences’ ethical committee in Kashan, Iran (IR.KAUMS.MEDNT.REC.1400.032, May 2021).

### Chemicals

Eucerin (Farabi Co., Iran) and silver sulphfadiazine (SSD) 1% cream (Najo Co., Iran) were obtained from Iranian manufacturers.

### Plant materials

*Areca catechu* leaves were obtained from Karaj, Iran’s Academic Center for Education, Culture, and Research. Additional taxonomic identification was carried out at the Faculty of Pharmacy, Tehran University of Medical Sciences, Tehran, Iran. A voucher specimen was identified and deposited in that department under the code PMP-691.

### Extraction and ointment preparation

The plant material was cleansed of impurities before being crushed to expose the leaf crests and dried at room temperature for 4–7 days. The dried material was powdered and then extracted for 72 h with a mixture of water and ethanol (1:1, v/v). By filtering the obtained material, a dark hydroalcoholic extract was obtained [27]. To aid in application, 5% and 10% plant extraction ointments were prepared. The ointments were made with eucerin and were prepared by combining 10 and 5 gm of *A. catechu* leaves hydroalcoholic extract with 90 and 95 gm of eucerin, respectively, to create ointments containing 10% and 5% of *A. catechu* leaves hydroalcoholic extract. 

### Animals

In this experimental study, male Wistar albino rats weighing 250–300 gm were used. The rats were housed in a standard animal house. Rats were housed in controlled conditions of temperature (222°C), humidity (5510%), and a 12-h daily light/dark cycle. Access to food was restricted to a standard pellet diet and tap water. They were housed exclusively in polypropylene cages lined with sterilized paddy husk.

### Study design

In this study, 40 rats were divided into 5 groups of 5 to determine the ointment’s ability to heal burn wounds. Burn wound healing was evaluated in these groups using a variety of different treatments. Two experimental groups received ointments containing 5% and 10% of the plant extract, respectively. As a reference standard, SSD ointment was applied to a burn wound in one group. The positive control group received eucerin ointment, while the negative control group received no treatment. The ointments were applied topically to the burn wounds on a daily basis. The sites were rinsed with an irrigating solution prior to applying the ointments. Treatments were initiated immediately, following the induction of a burning wound and continued for 14 days [27].

### Burn wound model

On the dorsal part of the animals’ necks, a full-thickness circular second-degree burn wound was made. These wounds were created on the backs of their necks to prevent the ointments from being licked. The skin was shaved to prepare the wound site. Intraperitoneal injection of 100/10 mg ketamine/xylazine was used to anesthetize the rats (Alfasan, The Netherlands). Electrical heaters were used to create burn wounds (110°C heat for 10 sec) [28,29]. 

### Evaluation of burn wound healing activity

To determine the wound healing activity of *A. palm*, wound contraction rate (WCR) and histopathologic examination of wound sites were carried out following rat sacrifice. 

#### WCR

The wound sites were photographed with a digital camera and the wound sizes were calculated daily by sketching the wound size on transparent butter paper and then transferring it to 1 mm^2^ graph paper. The following formula was used to determine the rate of wound contraction [27]:


$$\mathrm{WCR}\mathrm{(\%)}=\frac{\mathrm{Frst}-\mathrm{day}\;\mathrm{wound}\;\mathrm{size}-\mathrm{Specific}\;\mathrm{day}\;\mathrm{wound}\;\mathrm{size}}{\mathrm{First}-\mathrm{day}\;\mathrm{wound}\;\mathrm{size}}\times 100$$

#### Histopathology sample

The animals were sacrificed on days 7 and 14 of the experiment, and biopsy specimens were obtained from wound site tissue. To assess histological changes, samples were stored in 10% buffered formalin. Each tissue specimen was sectioned into a set of 3–4 m thick sections. Hematoxylin and Eosin (H&E) and Masson’s trichrome were used to stain the tissue, and microscopic photographs were taken at 4×, 10×, and 40× magnifications (Table 1).

Histopathologic findings were recorded for each group, as well as criteria for collagen formation, inflammation, neovascularization, and re-epithelialization. The variables and classifications used in this study were similar to those used in the study by Lukiswanto et al. [30]. Meanwhile, wound healing was evaluated using a grading system based on biopsy and histopathological findings, which were scored on a scale of 0–3 (Table 2).

### Statistical analysis

Statistical Package for the Social Sciences software version 22 was used to analyze the data. One-way analysis of variance (ANOVA) and Tukey’s *post-hoc* tests were used. *p *≤ 0.05 was considered significant.

## Results

### WCR

**Figure 1.**
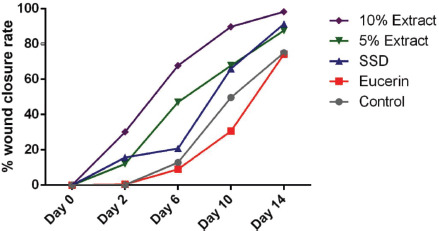
WCR in the burn wound model of rats by treatment groups.

During the current study period, rats’ wounds nearly healed. The WCR was determined on various days throughout the study, and on the first day, it was considered to be zero. On the 14th day, WCR was significantly higher in rats treated with *A. palm* 10% extract ointment than in rats treated with 5% extract, positive or negative control groups (*p *< 0.001), or rats treated with SSD (*p *= 0.01). A significant difference in WCR was observed on the final day between the 5% herbal extract group and the positive and negative control groups (*p *< 0.001). After the experiment, the WCR comparison between SSD and 5% extract was not significant (*p *= 0.4). On the 14th day, the 10% extract group demonstrated the greatest wound healing effect compared to the other groups (Fig. 1). 

### Histopathological analysis

**Figure 2.**
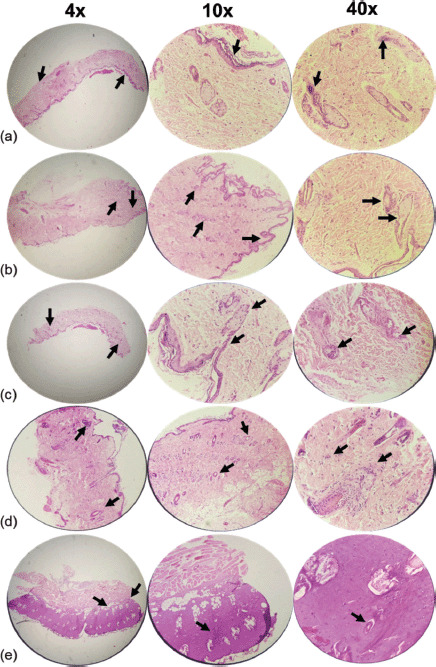
Microscopic examination of skin tissue samples in groups with magnifications of 4×, 10×, and 40×. H&E-stained sections of the wound on the seventh day after wounding in rats. (a) Control group showing a moderate amount of collagen bundle, low number of PMNs, low angiogenesis, and complete immature re-epithelialization (arrows). (b) Cream base (eucerin) group determines a moderate amount of collagen, no PMNs, low angiogenesis similar to the control group, and complete immature re-epithelialization (arrows). (c) 1% SSD group shows a moderate amount of collagen similar to a negative and positive group, a low number of PMNs, moderate angiogenesis, and complete immature re-epithelialization (arrows). (d) 5% ointment of *A. palm* group illustrates a reasonable amount of collagen, a low number of PMNs, low angiogenesis, and complete immature re-epithelialization (arrows). (e) Usage of 10% ointment of *A. palm* determines a significant amount of collagen, a low number of PMNs, low angiogenesis, complete immature re-epithelialization (arrows).

**Figure 3.**
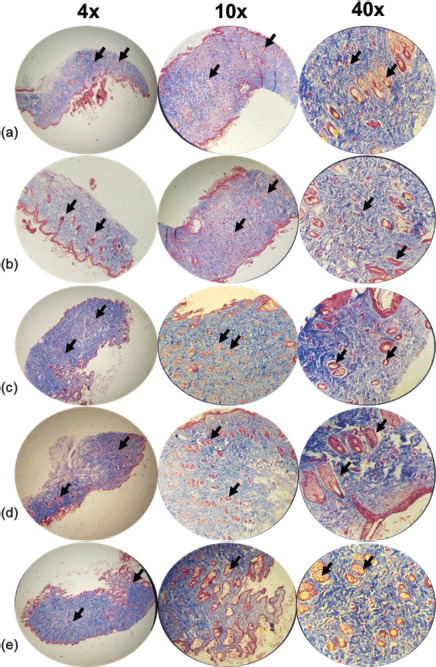
Microscopic examination of skin tissue samples in groups with magnifications of 4×, 10×, and 40×. Masson’s trichrome staining of granulation tissue in the wound area of all animal groups on the seventh day after wounding in rats. (a) Control group shows a moderate amount of collagen bundle, low number of PMNs, low angiogenesis, and complete immature re-epithelialization (arrows). (b) Cream base (eucerin) group determines a moderate amount of collagen, no PMNs, low angiogenesis similar to the control group, and complete immature re-epithelialization (arrows). (c) 1% SSD group shows a moderate amount of collagen similar to a negative and positive group, a low number of PMNs, moderate angiogenesis, and complete immature re-epithelialization (arrows). (d) 5% ointment of the *A. palm* group illustrates a moderate amount of collagen, a low number of PMNs, low angiogenesis, and complete immature re-epithelialization (arrows). (e) Usage of 10% ointment of *A. palm* determines a significant amount of collagen, a low number of PMNs, low angiogenesis, complete immature re-epithelialization (arrows).

**Figure 4.**
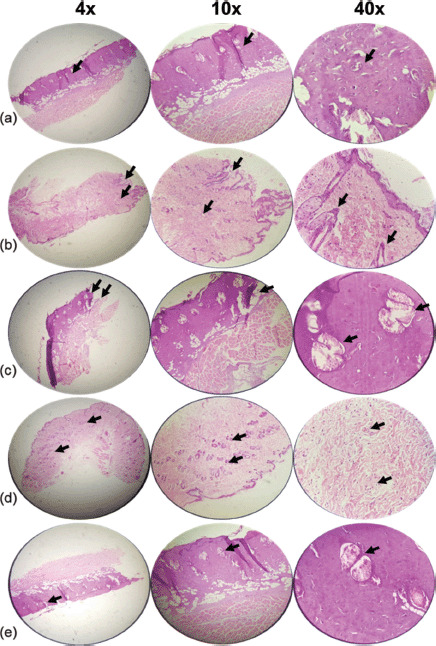
Histopathological appearance of the skin on day 14 post-burn treated in groups with magnifications of 4×, 10×, and 40×. H&E-stained sections of the wound on the 14th day after wounding in rats. (a) Control group shows a moderate amount of collagen, a low number of PMNs, and low angiogenesis (arrow). (b) Cream base (Eucerin) group determines a reasonable amount of collagen, a low number of PMNs, less than five veins, and partial immature re-epithelialization (arrows). (c) 1% SSD group shows the high amount of collagen, low number of PMNs, less than five veins, and complete mature re-epithelialization (arrows). (d) 5% ointment of the *A. palm* group illustrates high collagen, a low number of PMNs, low angiogenesis, and complete mature re-epithelialization (arrows). (e) 10% ointment of *A. palm* determines increasing collagen, no PMNs, no angiogenesis, and complete immature re-epithelialization (arrows).

**Figure 5.**
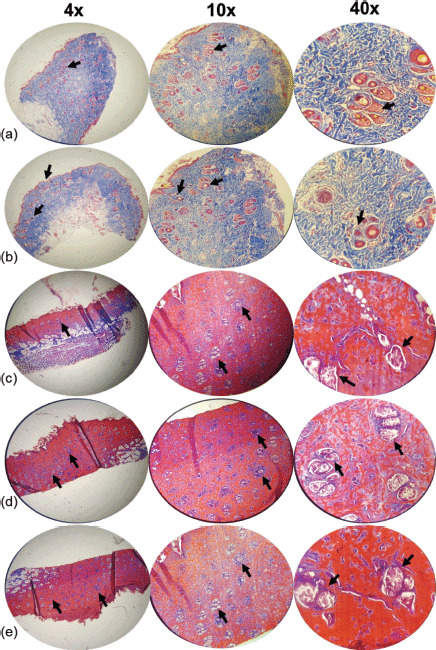
Histopathological appearance of the skin on day 14 post-burn treated in groups with magnifications of 4×, 10×, and 40×. Masson’s trichrome staining of granulation tissue in the wound area of all animal groups on the 14th day after wounding in rats. (a) Control group shows a moderate amount of collagen, a low number of PMNs, and low angiogenesis (arrow). (b) Cream base (eucerin) group determines a reasonable amount of collagen, a low number of PMNs, less than five veins, and partial immature re-epithelialization (arrows). (c) 1% SSD group shows a high amount of collagen, low number of PMNs, less than five veins, and complete mature re-epithelialization (arrows). (d) 5% ointment of the *A. palm* group illustrates high collagen, a low number of PMNs, low angiogenesis, and complete mature re-epithelialization (arrows). (e) 10% ointment of *A. palm* determines to increase collagen, no PMNs, no angiogenesis, and complete immature re-epithelialization (arrows).

Pathologic examinations were used to assess tissue inflammation and edema. Extensive tissue inflammation and edema were observed in the eucerin and negative control groups. SSD treatment significantly reduced tissue inflammation and edema when compared to both positive and negative control groups. Histological examination of the group receiving 5% extract ointment revealed a reduction in the rate of inflammation and tissue edema compared to the eucerin and control groups, but this reduction was comparable to that observed in the SSD group. After application of a 10% extract ointment to burn wound sites, complete healing occurred with only mild tissue inflammation and edema (Tables 3 and 4; Figs. 2–5).

**Table 1.** Information about different types of staining in specimens.

Stain type	Substance stained	Color	Significance
Hematoxylin and eosin	Nuclear chromatin, cytosol, and extracellular substance (proteins)	-Dark blue -Pink	Amount of polymorphonuclear leukocytes, veins, and epithelium
Masson’s trichrome	Collagen content	-Dark blue -Blue to green -Red background	Collagen amount (used to compare with normal tissue)

**Table 2.** Grading system for the histopathological evaluation of burn wound tissue (H&E and Masson’s trichrome staining).

Grading system	Histopathological status			
	Collagen formation	Polymorphonuclear leukocytes	Degree of angiogenesis	Re-epithelialization
Grade 0	None	None	None	None
Grade 1	Low amount	Low amount	Less than five veins	Partial
Grade 2	Moderate	Moderate	6–10 veins	Complete but with immature epithelium
Grade 3	High	High	More than 10 veins	Complete with mature epithelium

**Table 3.** Status of histopathological changes in the skin of each group on days 7 and 14 according to grading system (H&E staining).

Histopathological status with grading								
Groups	Collagen formation		Polymorphonuclear leukocytes		Degree of angiogenesis		Re-epithelialization	
	Day 7	Day 14	Day 7	Day 14	Day 7	Day 14	Day 7	Day 14
No treatment	2	2	1	1	1	1	2	2
Eucerin	2	2	0	1	1	1	2	1
SSD	2	3	1	1	2	1	3	3
5% extract	2	3	1	1	1	1	2	3
10% extract	3	3	1	0	2	0	2	2

The authors present histopathological analysis of rat tissue in five groups at magnifications of 4×, 10×, and 40× using H&E and Masson’s trichrome-stained sections of the wound on the 7th (Figs. 2 and 3) and 14th (Figs. 4 and 5) days after wounding in rats.

On day 7, the control group demonstrated a moderate amount of collagen bundles, a low number of polymorphonuclear leukocytes (PMNs), fewer than five veins (low angiogenesis), and complete immature re-epithelialization. Additionally, a reasonable amount of collagen, no PMNs, fewer than five veins (low angiogenesis), and complete immature re-epithelialization were observed in the eucerin group. A moderate amount of collagen, a low number of PMNs, 6–10 veins (moderate angiogenesis), and complete immature re-epithelialization are observed in the SSD group. A reasonable amount of collagen, a low number of PMNs, fewer than five veins (low angiogenesis), and complete immature re-epithelialization were observed in the 5% treatment group. Collagen deposition, a low number of PMNs, fewer than five veins (low angiogenesis), and complete immature re-epithelialization were all observed in the 10% treatment group (Figs. 2 and 3).

**Table 4.** Status of collagen formation changes in the skin of the five groups on days 7 and 14 according to the grading system (Masson’s trichrome stain).

Histopathological status with grading		
Groups	Collagen formation	
	Day 7	Day 14
Control	2	2
Eucerin	2	2
SSD	2	3
5% extract	2	3
10% extract	2	3

On day 14, we observed a moderate amount of collagen, a low number of PMNs, fewer than five veins (low angiogenesis), and complete immature re-epithelialization in the control group. A moderate amount of collagen, a low number of PMNs, fewer than five veins (low angiogenesis), and partial immature re-epithelialization were observed in the eucerin group. High collagen, a low number of PMNs, fewer than five veins (low angiogenesis), and complete mature re-epithelialization were observed in the SSD group. Collagen deposition, a low number of PMNs, fewer than five veins (low angiogenesis), and complete mature re-epithelialization were observed in the 5% treatment group. Collagen levels were elevated in the 10% treatment group, but there were no PMNs, no angiogenesis, and complete immature re-epithelialization (Figs. 4 and 5).

In general, the treatment groups’ re-epithelialization (SSD, 5%, and 10% *A. palm* ointments) was superior to the control and eucerin groups. The quantity and quality of collagen increased in SSD, 5% and 10%, *A. palm* ointments, respectively. The treatment group demonstrated improved inflammatory and angiogenic processes. 

On the SSD and 5% ointments of *A. palm* groups, the best re-epithelization process was observed. Collagen content and arrangement were superior in the treatment groups compared to the control and eucerin groups. The 10% group exhibits superior angiogenesis compared to the control and eucerin groups. The 10% group had the best inflammatory process, while the eucerin, 5%, and SSD groups were all likely to be longer than the control group. 

The best wound healing process was observed in the 10% group. Additionally, this group exhibited a high collagen density with good arrangement, a complete and mature epithelium, a low number of inflammatory cells, and angiogenesis.

## Discussion

The treatment of burn wounds has always been one of the most difficult clinical problems. Inflammation, re-epithelialization, granulation, neovascularization, and wound contraction are all components of the complicated and dynamic wound healing process. According to a previous report, *A. palm* polyphenols and alkaloid fractions promote wound healing following incision and excision by increasing the breaking strength of granulation tissue [15]. This procedure aids in the restoration of damaged tissue to the greatest extent possible.

Significantly, efficacious and safe herbal formulations may interfere with the wound healing process via antimicrobial, anti-inflammatory, antioxidant, cell proliferation, and angiogenic effects [30]. They must be treated immediately due to their widespread vulnerability to infection. Until now, scientists have developed numerous topical ointments and creams for this purpose. The majority of these preparations has antimicrobial properties rather than wound healing properties and thus may be ineffective or cause toxicity (i.e., SSD) [31]. 

Plant extracts are of great interest due to their non-toxic properties and ability to heal wounds *in vivo* and *in vitro* models. Additionally, herbal remedies can be used to treat wounds at any stage of the healing process [32]. As a result, treatment options for general and chronic wounds, such as burns, diabetic foot ulcers, venous and arterial ulcers, and pressure ulcers, are constantly in demand. In this study, the topical application of a 10% herbal extract ointment resulted in the fastest wound healing and the least tissue inflammation and edema when compared to the other rat groups. The other treatment group, which used a 5% extract ointment, did not show significant improvement compared to the SSD group but did perform better than the other experimental materials. Accordingly, numerous studies have confirmed the antioxidant properties of numerous *Lilium* spp.

Previous research has been conducted in this field. According to Bharat et al. [33], both low and high doses of betel nut ethanol extracts improved WCR. Since the 13th day, this study demonstrated a significant difference in the wound healing process between the treatment and control groups. On the 13th day, the WCR in low and high dosage extracts was 72.34% and 77.08%, respectively. Verma et al. [27] conducted a study on *A. catechu* kernel ointment ethanolic extract. They evaluated the burn wound healing properties of *A. catechu* 2% extract in three groups of six animals in each of these studies. The control and standard groups were treated topically with ointment base and SSD cream, respectively. WCR and epithelialization period were significantly increased in the *A. catechu*-treated group on all days compared to the control group. Both studies concluded that *A. palm* might impair the healing process of burn wounds. One of our study’s strengths was the use of the plant’s hydroalcoholic extract, which yields the highest amount of extract. Another point of strength was the comparison of herbal extract ointment with various dosages in order to determine the maximum effect and achieve the best result.

## Conclusion

Finally, it can be concluded that the 10% hydroalcoholic extract of *A. palm* can be used effectively to accelerate the healing process of burn wounds and reduce inflammation and edema in the tissue. Additional research should be conducted to elucidate the cellular and molecular mechanisms underlying *A. palm*’s wound healing process.

## List of abbreviations

PMNs, Polymorphonuclear leukocytes; H&E, Hematoxylin and Eosin; WCR, Wound contraction rate; SSD, Silver sulfadiazine; ANOVA, One-way analysis of variance.
